# High street retail environment interventions and their theorised impacts on health and wellbeing: A scoping review

**DOI:** 10.1371/journal.pone.0312826

**Published:** 2024-11-14

**Authors:** Chiara Rinaldi, Elizabeth McGill, Mark Petticrew, Cécile Knai, Matt Egan

**Affiliations:** 1 Department of Public Health, Environments and Society, London School of Hygiene and Tropical Medicine, London, United Kingdom; 2 Department of Health Services Research and Policy, London School of Hygiene and Tropical Medicine, London, United Kingdom; University of Amsterdam, NETHERLANDS, KINGDOM OF THE

## Abstract

Health outcomes are influenced by social and environmental determinants of health. As places where people work, live, meet and consume, high street retail environments are influential in shaping health. In recent decades, high streets have been in decline, prompting policies to revitalise retail environments and support local businesses, particularly in European and North American countries. The aim of this scoping review was to systematically map evidence on retail environment interventions, to gain a deeper understanding of the current evidence base assessing their possible health and wellbeing impacts. The objectives were to identify different types of interventions and the outcomes they address; and the mechanism through which interventions are theorised to influence health and equity. Peer-reviewed studies were identified through academic databases (MEDLINE, Embase, EconLit, Web of Science and Social Policy and Practice) using relevant search terms. Additional (grey) literature was identified using citation scanning and online searches. Studies were eligible if they evaluated interventions with a significant focus on supporting the retail environment, reported on at least one health and wellbeing outcome and were written in English. Relevant data were extracted and presented descriptively. An interpretive approach was taken to analyse theories of change. The searches identified 53 peer-reviewed studies and nine grey literature reports. Interventions were categorised as follows: area-based initiatives, business improvement districts, business incentives, and demand-side incentives. Studies predominantly evaluated impacts on social and environmental determinants of health. Some studies measured impacts on self-rated (mental) health, physical activity and food consumption and purchasing. Studies reported evidence of both improved and worsening outcomes. Theories of change were often under-specified and reductionist, lacking a clear understanding of the complex systems in which interventions take place. Future interventions could benefit from more comprehensive theories of change that meaningfully integrate economic, and health and wellbeing outcomes. This requires intersectoral collaboration.

## Introduction

Population health outcomes are partly shaped by people’s social and economic circumstances and the environments in which they spend their lives [1, 2]. These factors are referred to as the social and environmental determinants of health and include stable employment, education, safe housing, access to healthy food and other necessities, social integration, community safety and the neighbourhood environment. In urban areas, high streets are important places where people meet, work and live [1]. High streets are also retail environments where products and services are promoted for sale, therefore influencing patterns of consumption. Acting as physical, social and commercial environments, characteristics of local retail environments can influence the health of residents and visitors in multiple, sometimes counteracting, ways [3–5]. High streets are essential to local economies as a major source of employment and income, and often act as centres for social and community activity. However, they can also provide easy access to unhealthy commodities and the outlets that promote them, including foods high in fat, salt and sugar, alcohol, tobacco and gambling outlets [6]. The wide availability of these products and services has been found to contribute to increased use or consumption, which may lead to a variety of negative health outcomes in the long term, including mental health outcomes, obesity, diabetes and cardiovascular disease [7–9]. Retail environments in more socio-economically disadvantaged areas tend to have greater clustering of products and services that can be damaging for health, thereby contributing to health inequalities [6, 10].

In recent decades, high streets in many high income countries (particularly in Europe and the United States) have been in decline. There are multiple reasons for this [11]. Long-term trends such as an increase in out-of-centre shopping developments and online retail have changed consumer purchasing behaviour away from the traditional high street [12, 13]. Increasingly, high streets are serving different purposes, such as housing, professional services, leisure and community building [14, 15]. The retail environment is also influenced by demographic changes, such as an ageing population and changes in urban living [16]. These trends have been exacerbated by economic downturn, inflation and, more recently, the COVID-19 pandemic [13, 17], which have contributed to the closure of retail businesses, area degradation, and a loss of footfall and social cohesion [3, 4, 18]. The impacts of declining high streets disproportionally affect already disadvantaged cities and towns, and people that rely more on their local area, for example due to having decreased access to transport to reach out-of-centre retail [15]. Governments in many countries have responded through initiatives aimed at revitalising retail environments and supporting local economies [11, 19–21]. Such initiatives can be primarily focused on increasing income for local businesses and attracting new commercial activity. Other initiatives have a broader focus on improving economic, social and community outcomes for people living in areas in economic and physical decline [22]. Such initiatives include improving employment outcomes, enhancing living environments, encouraging active travel and promoting social cohesion [22]. Depending on the aims and objectives of interventions and the interests that they primarily serve, they can help improve health and wellbeing outcomes indirectly. For instance, by supporting local economies on which many livelihoods depend [23]. However, failing to consider the wider implications of policies aimed at supporting the economic viability of high streets could have negative impacts on population health and health equity.

Existing literature and systematic reviews tend to focus on the impacts of interventions designed from a public health perspective. This includes reviews on supermarket interventions (e.g. interventions addressing in-store marketing, pricing and product placement) [24–27], interventions addressing the food offered in (fast) food outlets [28–30] and other healthy food environment interventions [31]. Other reviews have focused on urban regeneration interventions aimed at making the built environment more health-promoting, including housing regeneration programmes and the introduction of urban green spaces [32–39]. Less evidence exists on the social and public health impacts of broader policies and interventions addressing the retail environment, such as financial or planning interventions to improve the economic viability and vitality of high streets. This is important, as many interventions that do not (solely) have health as the main intended outcome have implications for health and inequalities through the social and environmental determinants of health [1, 23].

Reviews suggest the health impacts of such interventions are often poorly evidenced. For example, a systematic review by Mah and colleagues [40] examining the role of public policy in food retail environment interventions identified a lack of research that goes beyond the effects of interventions within the health sector. There was limited evidence acknowledging the wider policy environments (e.g. fiscal policies, economic business support, health-promoting regulation) that are required for the success of the intervention. Much of the evidence is focused on interventions targeting premise-level environments (e.g. inside shops and restaurants) rather than on neighbourhood retail environments such as high streets. Mah et al.’s [40] review exclusively focuses on food retail environments and excludes literature on interventions not specifically aimed at promoting health.

This scoping review aimed to systematically map evidence on interventions that aim to revitalise retail environments, to gain a deeper understanding of the evidence base assessing their possible impacts on health and wellbeing. The objectives were to identify the main types of interventions and their aims, reported health and wellbeing outcomes and other outcomes relevant to social determinants of health, their theories of change and to identify any knowledge gaps.

## Methods

This scoping review follows guidance by Arksey and O’Malley [41], updated by Levac et al. [42]. Their framework sets out six steps to conducting a scoping review: 1) identifying the research question; 2) identifying relevant studies; 3) study selection, 4) charting the data; 5) collating, summarizing, and reporting results; and 6) consultation.

### Identifying the research question

As outlined above, the research question for this scoping review are *“What are the main types of retail environment interventions in the literature*, *and what health and wellbeing outcomes do they address*?*”* and *“What are the mechanisms by which retail environment interventions are theorised to promote or hinder health (equity) and wellbeing*?*”*.

### Identifying relevant studies

Studies were identified through electronic databases MedLine, Embase, EconLit, Social Policy and Practice (through the Ovid platform) and Web of Science and in November-December 2022. Searches were completed using key words and terms for high street/urban revitalisation initiatives, health and wellbeing outcomes and evaluations (the full search strategy can be found in the S1 Appendix). No date and geographic limits were applied. Further peer-reviewed publications were identified through reference screening. A grey literature search was performed to identify evaluation reports (e.g. those commissioned for government interventions) that were not published in a peer-reviewed journal. Grey literature was identified by searching Google and Google Scholar search engines, the Open Grey database and selected government websites using similar key words (S1 Appendix).

### Study selection

To be eligible, studies were required to 1) evaluate interventions that aim to support or revitalise the retail environment; 2) report on at least one health and wellbeing related outcome (including social and environmental determinants of health and health inequalities); 3) be written in English. For the purpose of this review, ‘interventions’ include any type of initiative, policy or programme aimed at improving or supporting the neighbourhood retail environment (as opposed to the consumer retail environment, e.g. in-store environment), with or without explicit health and wellbeing objectives. Interventions at local, regional and national level were eligible. Broader area-based interventions were only included if they had a significant focus on retail (business) support and investment. Due to extensive previous research and systematic reviews on the health and wellbeing implications of urban (green) infrastructure [32–36] and housing interventions [37, 38], studies of area based initiatives that focused primarily on housing or green space were excluded. Health and wellbeing outcomes were conceptualised broadly as including direct physical and mental health outcomes, health behaviours (e.g. food consumption and purchasing) as well as outcomes related to the social and environmental determinants of health (e.g. employment and income, crime and safety, access to healthy food). Both peer-reviewed and grey literature reports available in full-text were eligible for inclusion, while abstracts, protocols, editorials and webpages were excluded. Theoretical modelling studies that did not evaluate an existing intervention, systematic reviews and health impact assessments (HIA) were also excluded from this review.

Titles and abstracts were initially screened for inclusion by CR, after which a full-text assessment was performed. A second reviewer (EM) independently screened 10% of the reports at each stage. Disagreements between reviewers were resolved through discussion, and a third opinion by ME.

### Charting the data

A data charting template [42] was created to extract information from included studies and reports. The template was refined throughout the data charting process to capture all relevant information. This included data on the study aims; country and context; intervention type, aims and target population; intervention ownership; study design and methods; data sources; primary health and wellbeing outcomes and outcome type; theory/mechanisms of change; and a summary of findings. Study findings were not analysed in depth (e.g. through meta-analysis), but were categorised as positive, limited positive, neutral, limited negative and negative for health and wellbeing outcomes. Evidence was classed as “limited” positive or negative if only some findings were positive/negative, but not enough evidence was reported to deem the intervention effective in improving/worsen the outcomes of interest. Data were charted by CR and reviewed by EM (S1 Dataset).

In addition, an interpretive approach was used to analyse mechanisms by which interventions were theorised to promote or hinder health and wellbeing outcomes and equity (theories of change). This synthesis draws on the Critical Interpretive Synthesis (CIS) approach [43]. In contrast to many systematic reviews, CIS draws on a qualitative research tradition of interpreting data. It does not only aggregate and summarise available information, but interprets findings with the aim of developing new theory and concepts [43]. CIS reviews have a distinct study identification process that is not based on a highly specified research question, search strategy and inclusion criteria [43]. This is done to be able to draw on a more diverse and comprehensive sample of evidence. This aspect was not applied in this review, which followed a structured scoping review methodology [42, 44]. Instead, this review draws on CIS’s interpretive approach to data charting and synthesis. In particular, the concept of ‘synthesising argument’ was used. A synthesising argument is an argument or narrative that is generated through analysis of evidence across the included studies, drawing on primary evidence as well as the interpretations and theories used by study authors to interpret their study findings [43]. Based on an initial reading of the included studies, themes and categories were assigned to the data. These were based on theories of change as well as the interpretations and critiques by the authors of included studies (who were typically not involved in the design of the interventions they evaluated). Information about theories of change (description; type; theoretical framework; effects on wider retail environment; equity impacts; authors’ critique) was charted for each individual study (S1 Dataset). This information was based on the included studies identified in this scoping review and their (health and wellbeing) outcomes of interest, which may not represent the full overarching theory of change of the intervention reported elsewhere.

### Collating and summarising

The charted data is presented in the form of descriptive information of the studies and evaluated interventions, and a narrative summary of the interventions’ theories of change. This process was guided by the research questions of this review. The Preferred Reporting Items for Systematic Reviews and Meta-Analyses Extension for Scoping Reviews (PRISMA-ScR) checklist was used for reporting (see S1 Checklist) [44].

Ethical approval for this study was granted by the London School of Hygiene and Tropical Medicine Research Ethics Committee. Due to the data collection being secondary (published peer-reviewed and grey literature) informed consent was not necessary.

## Results

**Fig 1:**
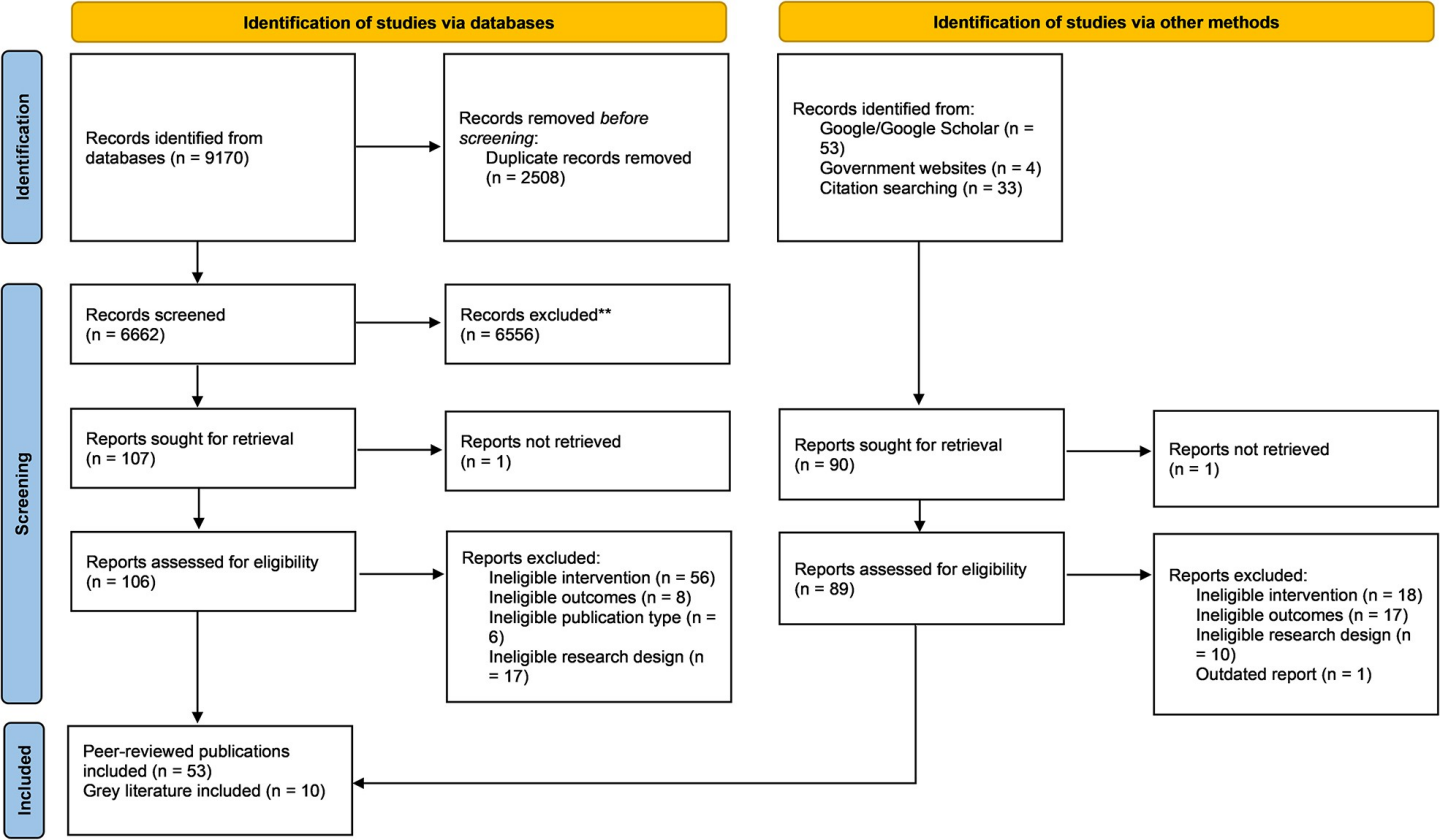
PRISMA flowchart.

The search strategy identified 6,662 peer-reviewed studies after removal of duplicates. An additional 90 studies were identified using alternative searches. After the screening process, 53 studies published in peer-reviewed journals and nine grey literature reports were included in this scoping review (see Fig 1). Characteristics about each of the evaluations can be found in Tables 1 and 2. Over three quarters of the evaluations were predominantly quantitative, including before/after evaluations (n = 17), cross-sectional studies (n = 8), quasi-experimental studies (n = 5), longitudinal studies (n = 23), one cohort study and one randomised controlled trial. However, eight peer-reviewed studies (15.1%) and 7 grey literature studies (77.8%) did not include a comparison area or group. This makes it difficult to attribute observed effects to these interventions, especially given the complex systems that the interventions aim to address. The identified studies evaluated interventions in the United States (US) (n = 43), United Kingdom (UK) (n = 17), Canada (n = 1) and Norway (n = 1). Almost half of the interventions (45.2%) took place in contexts described as ‘disadvantaged’ neighbourhoods or areas. In addition, 16 studies took place in unspecified urban areas, 12 in so-called ‘food deserts’ (areas with low access to fresh food), four in city centres, one in a rural area and one in an ‘ethnically diverse state’. Studies were not limited to the public health literature, given the broader scope of the interventions of interest. The majority of peer-reviewed studies were published in medical/public health (n = 18), urban planning (n = 13) and economics (n = 12) journals. Nutrition (n = 5), public policy (n = 2), law (n = 1) and criminology (n = 1) publications were also represented in the sample.

**Table 1:** Information from included peer-reviewed literature.

Authors (date)	Country; Context	Study aims	Intervention type	Intervention specified	Time frame	Intervention aims	Ownership	Outcome type	Health and wellbeing related outcomes	Intervention impact
**Clutter, Henderson & Haberman (2019) [45]**	United States; inner city retail area	Assess impacts on street block robbery	Business improvement district	Business improvement district in high streets of Cincinnati (Ohio), with an annual business-funded budget to fund supplemental public services in the area	1990 onwards	To revitalise urban commercial areas and increase commercial activity	Public-private partnership (businesses located in BID area and local government)	Social determinants of health	Street block robbery	Negative
**Brooks (2008) [46]**	United States; inner city retail area	Assess impact on crime rates	Business improvement district	Business improvement districts in Los Angeles, with an annual business-funded budget to fund supplemental public services in the area	1994 onwards	To revitalise urban commercial areas and make them attractive, safer, cleaner, and more marketable	Public-private partnership (businesses located in BID area and local government)	Social determinants of health	Robbery; burglary; theft; overall crime rate; less serious crime rate	Positive
**Cook & MacDonald (2011) [47]**	United States; inner city retail area	Assess impact on security						Social determinants of health	Robbery; assault; burglary; and auto theft	Positive
**MacDonald, Golinelli, Stokes & Bluthenthal (2010) [48]**	United States; inner city retail area	Assess impact on violent crime rates						Social determinants of health	Robbery; violent crimes	Positive
**MacDonald, Stokes, Grunwald & Bluthenthal (2013) [49]**	United States; inner city retail area	Assess impact on violent crime victimisation among adolescents						Social determinants of health	Self-reported adolescent violent victimization; robbery; threatening with weapon; beating; physical attack by group; witness of group assault	Neutral
**Sutton (2014) [50]**	United States; inner city retail area	Assess impact on retail sales and local employment	Business improvement district	Business improvement districts in New York City, with an annual business-funded budget to fund supplemental public services in the area	2000 onwards	To revitalise urban commercial areas and increase commercial activity	Public-private partnership (businesses located in BID area and local government)	Social determinants of health	Change in employment rates	Limited negative
**Han, Morcol, Hummer & Petersen (2017) [51]**	United States; inner city retail area	Assess impact on minor crime rates	Business improvement district	Business improvement districts in Philadelphia, with an annual business-funded budget to fund supplemental public services in the area	1998–2009	To revitalise urban commercial areas and increase commercial activity	Public-private partnership (businesses located in BID area and local government)	Social determinants of health	Nuisance crimes of graffiti; illegal dumping; disorderly conduct	Limited positive
**Hoyt (2005) [52]**	United States; inner city retail area	Assess impacts on criminal activity in and around commercial area	Business improvement district	Business improvement districts in Philadelphia, with an annual business-funded budget to fund supplemental public services in the area	2002 onwards	To revitalise urban commercial areas and increase commercial activity	Public-private partnership (businesses located in BID area and local government)	Social determinants of health	Stolen vehicles; theft from vehicles; thefts; burglaries; robberies; property crimes; drug-related crimes; disorderly conduct; quality-of-life crimes	Positive
**Oakley & Tsao (2006) [53]**	United States; Disadvantaged neighbourhoods	Assess impact on socio-economic outcomes for area residents.	Business incentives	Empowerment Zone (EZ) and Enterprise Community (EC) Initiative offering targeted funding and tax incentives to businesses in distressed areas (in Baltimore, Chicago, Detroit and New York City). Businesses need to create a strategic plan to create community partnerships, economic opportunity for residents and sustainable community development	1994 onwards	To attract public and private resources to revitalise distressed communities, and expand employment opportunities and alleviate poverty	Public-private partnerships (US Government and businesses)	Social determinants of health	Unemployment rate; residents below poverty line	Neutral
**Reynolds & Rohlin (2014) [54]**	United States; Disadvantaged areas	Assess impacts on local employment and poverty	Business incentives	Empowerment Zone (EZ) and Enterprise Community (EC) Initiative offering targeted funding and tax incentives to businesses in distressed areas		To attract public and private resources to revitalise distressed communities, and expand employment opportunities and alleviate poverty.	Public-private partnership (Regional government and local businesses)	Environment determinants of health	Local quality of life (based on quality of local amenities)	Limited positive
**Hanson (2009) [55]**	United States; Disadvantaged areas	Assess impacts on local employment	Business incentives	Enterprise Zones in Atlanta, Baltimore, Chicago, Detroit, New York and Philadelphia, providing incentives for businesses to locate or expand in targeted (economically deprived) areas	1985 onwards	To improve and regenerate economically deprived areas, and create new jobs for local residents, reduce unemployment and increase income	Public-private partnership (Regional government and local businesses)	Social determinants of health	Employment rate	Neutral
**Elvery (2008) [56]**	United States; Disadvantaged neighbourhood	Assess impacts on neighbourhood employment rates	Business incentives	Enterprise Zones in California and Florida providing incentives for businesses to locate or expand in targeted low-income areas. Incentives include property and income tax abatements, advantageous permitting and regulation, infrastructure improvements and tax credits for job creation	1986–1990	To improve and regenerate economically deprived areas, and create new jobs for local residents and reduce unemployment and income	Public-private partnership (Regional government and local businesses)	Social determinants of health	Neighbourhood employment rate	Limited negative
**Dowall (1996) [57]**	United States; Disadvantaged areas	Assess impacts on local employment	Business incentives	Enterprise Zones in California providing incentives for businesses to locate or expand in targeted low-income areas. Incentives include property and income tax abatements, advantageous permitting and regulation, infrastructure improvements and tax credits for job creation	1986 onwards	To improve and regenerate economically deprived areas, and create new jobs for local residents, reduce unemployment and increase income	Public-private partnership (Regional government and local businesses)	Social determinants of health	Employment rate; Employment growth	Neutral
**Neumark & Kolko (2010) [58]**	United States; Disadvantaged areas	Assess impacts on local employment			1985 onwards			Social determinants of health	Employment rate; Employment growth; Share of employees in low-wage industries	Neutral
**O’Keefe (2004) [59]**	United States; Disadvantaged neighbourhoods	Assess impacts on neighbourhood employment rates			1992–1999			Social determinants of health	Growth of employment; growth of monthly earnings	Limited positive
**Greenbaum & Engberg (2000) [60]**	United States; Disadvantaged areas	Assess impacts on local employment and poverty	Business incentives	Enterprise Zones in California, Florida, New Jersey, New York, Pennsylvania, and Virginia providing incentives for businesses to locate or expand in targeted (economically disadvantaged) areas.	1982 onwards	To improve and regenerate economically deprived areas, and create new jobs for local residents, reduce unemployment and increase income	Public-private partnership (Regional government and local businesses)	Social determinants of health	Unemployment rate; Poverty rate; Employment rate; Per capita income	Limited negative
**Bondonio & Engberg (2000) [61]**	United States; Disadvantaged areas	Assess impacts on local employment	Business incentives	Enterprise Zones in California, Kentucky, New York, Pennsylvania and Virginia providing incentives for businesses to locate or expand in targeted (economically disadvantaged) areas.	1983 onwards	To improve and regenerate economically deprived areas, and create new jobs for local residents, reduce unemployment and increase income	Public-private partnership (Regional government and local businesses)	Social determinants of health	Employment growth; unemployment rate	Neutral
**Lambert & Coomes (2001) [62]**	United States; Disadvantaged areas	Assess impacts on local employment and poverty	Business incentives	Enterprise Zones in Louisville providing incentives for businesses to locate or expand in targeted (economically disadvantaged) areas.	1982 onwards	To improve and regenerate economically deprived areas, and create new jobs for local residents, reduce unemployment and increase income	Public-private partnership (Regional government and local businesses)	Social determinants of health	Job growth; Unemployment rate; People below the poverty line; Employment rate; Household income	Negative
**Ham, Swenson, İmrohoroğlu & Song (2011) [63]**	United States; Disadvantaged areas	Assess impacts on local employment and poverty	Business incentives	Enterprise Zones, Empowerment Zones, and Enterprise Community Program. Providing incentives for businesses to locate or expand in targeted (economically disadvantaged) areas and hire local residents.	1982 onwards	To improve and regenerate economically deprived areas, and create new jobs for local residents, reduce unemployment and increase income	Public-private partnership (Regional government and local businesses)	Social determinants of health	Unemployment rate; Poverty rate; Employment rate; Fraction of households with income	Positive
**Freedman, Bell, Clark, Ngendahimana, Borawski, Trapl, Pike & Sehgal (2021) [64]**	United States; Food desert	Assess impacts on overall neighbourhood built and social food environment	Business incentives	Federal Healthy Food Financing Initiative (HFFI) providing financing assistance and incentives for the introduction of healthy food outlets in food deserts. This study focuses on the introduction of new centrally located food hub in an area with low food access in Cleveland. The food hub includes a catering service, healthy café, grocery store, farmers market, mobile market and on-site culinary job training and workshops	2015	To foster community economic development in a poverty-stricken area, including business development, job creation, community revitalization, and healthy food access through elimination of a food desert	Whole of society partnership (US government, local government, community development group, local foundation, NGOs and local businesses)	Food consumption and purchasing	Dietary behaviours; observed healthy food availability scores; perceptions of healthy food availability; social support for healthy eating	Neutral
**Sharpe, Bell, Liese, Wilcox, Stucker & Hutto (2020) [65]**	United States; Food desert	Assess impacts on diet, energy intake, BMI and perceived food environment						Food consumption and purchasing; Physical health	Fruit and vegetable consumption; daily energy intake; dietary quality; BMI; household food security; physical activity; self-efficacy; fast food intake; perceived neighbourhood food environment	Limited positive
**Dubowitz, Ghosh-Dastidar, Cohen, Beckman, Steiner, Hunter, Flórez, Huang, Vaughan, Sloan, Zenk, Cummins & Collins (2015) [66]**	United States; Food desert	Assess impact on diet, obesity (BMI), and perceived access to healthy food.	Business incentives	Federal Healthy Food Financing Initiative (HFFI) providing financing assistance and incentives for the introduction of healthy food outlets in food deserts. This study focuses on the introduction of a new full-service supermarket in Pittsburgh, Pennsylvania	2013	Improve access to healthy foods in underserved areas, create and preserve quality jobs, and revitalize low-income communities.	Public-private partnership (US Government, local government and local businesses)	Food consumption and purchasing; Physical health	Diet quality; daily energy intake; BMI; perceived access to healthy food	Limited positive
**Ghosh-Dastidar, Hunter, Collins, Zenk, Cummins, Beckman, Nugroho, Sloan, Wagner & Dubowitz (2017) [67]**	United States; Food desert	Assess impacts on geographic supermarket access, availability of healthy and unhealthy foods, and overall food prices.						Environmental determinants of health	Proximity to food store; fruit and vegetable availability; healthy food availability; unhealthy food availability; fast food availability; food prices	Limited positive
**Singleton, Li, Odoms-Young, Zenk & Powell (2019) [68]**	United States; Food desert	Assess impacts on fruit and vegetable availability in local food environment	Business incentives	Federal Healthy Food Financing Initiative (HFFI) providing financing assistance/grants for the introduction of healthy food outlets. This study focuses on a supermarket in Illinois.	2015	Improve access to healthy foods in underserved areas, create and preserve quality jobs, and revitalize low-income communities.	Whole of society partnership (US government, local government, Illinois Fresh Food Fund, local businesses)	Environment determinants of health	Food and beverage availability; Interior and exterior store marketing	Limited positive
**Elbel, Moran, Dixon, Kiszko, Cantor, Abrams & Mijanovich (2015) [69]**	United States; Disadvantaged neighbourhoods	Assess impacts of household food availability and children’s diets	Business incentives	Food Retail Expansion to Support Health (FRESH) program in New York City, lowering costs of owning, leasing and renovating supermarket retail space (in underserved, low-income) areas via tax and zoning incentives, for large supermarkets selling significant healthy food options	2011 onwards	Improving fruit and vegetable access in deprived neighbourhood and stimulate the local economy and employment	Public-private partnership (NYC Economic Development Corporation, NYC Department of Health and Mental Hygiene and local businesses)	Food consumption and purchasing	Household food availability; dietary intake	Neutral
**Rogus, Athens, Cantor & Elbel (2018) [70]**	United States; Disadvantaged neighbourhoods	Assess impacts on household food availability and dietary intake						Food consumption and purchasing	Household food availability; dietary intake	Positive
**Elbel, Mijanovich, Kiszko, Abrams, Cantor, & Dixon (2017) [71]**	United States; Disadvantaged neighbourhood	Assess impacts on food purchasing and dietary habits in adults						Food consumption and purchasing	Food consumption; changes in food consumption; household food availability; fruit and vegetable purchasing; food shopping behaviour	Limited positive
**Rummo, Sze & Elbel (2022) [72]**	United States; Disadvantaged neighbourhoods	Assess impacts on children’s and adolescents’ weight status						Physical health	BMI	Limited positive
**Wrigley, Warm & Margetts (2003) [73]**	United Kingdom; Food desert	Assess changes in food consumption	Area-based initiative	Introduction of a large superstore and 10 smaller units in centre of deprived area in Seacroft, Leeds under regeneration partnership between food retailer and local government	2000 onwards	To improve and regenerate town centre; increase food access for residents living in deprived neighbourhood with poor food retail access; and create employment	Whole of society partnership (local government, major food retailer, property developers, employment services agency, shop workers’ unions and local family centre)	Food consumption and purchasing	Dietary quality (change); Fruit and vegetable consumption	Positive
**Wrigley, Warm, Margetts & Whelan (2002) [74]**	United Kingdom; Food desert	Assess changes in food consumption						Food consumption and purchasing	Dietary quality (change); Fruit and vegetable consumption	Positive
**Cummins, Findlay, Higgins, Petticrew, Sparks & Thomson (2008) [75]**	United Kingdom; Food desert	Assess changes in diet, self-reported health, and perceptions of neighbourhood	Area-based initiative	Introduction of hypermarket in deprived area in Glasgow under area regeneration partnership	2001 onwards	Stimulating retail-led area regeneration, providing long term training and job security, and improving food access in deprived area with poor food access.	Public-private partnership (Tesco St Rollox Partnership between major food retailer, Glasgow Chamber of Commerce, local training college, and regeneration companies)	Food consumption and purchasing; Mental health and wellbeing; Physical health	Fruit and vegetable consumption; self-rated health score; psychological wellbeing score	Limited positive
**Cummins, Petticrew, Higgins, Findlay & Sparks (2005) [76]**	United Kingdom; Food desert	Assess impacts on fruit and vegetable consumption, self-reported, and psychological health						Food consumption and purchasing; Mental health and wellbeing; Physical health	Fruit and vegetable consumption; self-reported health; psychological health	Limited positive
**Cummins, Findlay, Petticrew & Sparks (2005) [77]**	United Kingdom; Food desert	Assess effects on existing retail structure, food choice, physical and economic food access, and employment						Environmental determinants of health; Social determinants of health	Change in food environment; amount of local fruit and vegetable retailers; economic food access; neighbourhood employment	Limited positive
**Chaput, Mercille, Drouin & Kestens (2018) [78]**	Canada; Disadvantaged neighbourhood	Assess impacts on physical access and fruit and vegetable consumption	Business incentives	Introduction of new fruit and vegetable market near subway station, selling locally produced and affordable food with funding from the Montreal Public Health Department	2016	Improving fruit and vegetable access and affordability in deprived neighbourhood and stimulate the local economy	Public community partnership (Social economy enterprise Y’a QuelQu’un l’aut’bord du mur and Montreal Public Health Department)	Food consumption and purchasing	Fruit and vegetable consumption; perceived access to fruits and vegetables	Positive
**Sadler, Gilliland & Arku (2013) [79]**	United States; Food desert	Assess the impact on price and availability of food	Area-based initiative	Introduction of new independent food retailers in the centre of a deprived area in Flint, Michigan, with financial assistance from NGO agencies, tax incentives and commercial revitalization deductions	2010 onwards	Contributing to retail-led area regeneration	Whole of society partnership (Local government, local businesses and NGO agencies)	Food consumption and purchasing	Fruit and vegetable consumption; nutrition quality; food security	Limited negative
**Sadler, Gilliland & Arku (2013) [80]**	United States; Food desert	Assess the introduction of a new food retailer on food consumption and security						Environmental determinants of health	Food prices; proximity to grocery store	Positive
**Clark, Smuk, Cummins, Eldridge, Fahy, Lewis, Moore, Smith, Taylor & Stansfeld (2018) [81]**	United Kingdom; Disadvantaged neighbourhood	Assess the impacts on self-rated mental health	Area-based initiative	London 2012 Olympics related urban regeneration including introduction of retail and leisure centre including retail space, office and business space and community space. The intervention also includes the creation of the Olympic Park (green space, sports facilities), housing and built environment improvements (2011–2014)	2011–2014	To leave a lasting legacy for the residents of east London through improvements to infrastructure and housing, stimulating economic development and aiming to ‘inspire a generation’ to be more physically active.	Public-private partnership (UK Government)	Mental health and wellbeing	Psychological wellbeing score; depressive symptoms	Limited positive
**Cummins, Clark, Lewis, Smith, Thompson, Smuk, Stansfeld, Taylor, Fahy, Greenhalgh & Eldridge (2018) [82]**	United Kingdom; Disadvantaged neighbourhood	Assess the impacts on employment, health behaviours, self-rated physical and mental health						Physical activity; Mental health and wellbeing	Psychological wellbeing score; depressive symptoms; physical activity levels; sedentary behaviour	Limited positive
**Van Leuven (2022) [83]**	United States; rural area	Assess impacts on local employment and establishments	Area-based initiative	Main Street Program; providing resources and practical knowledge to revitalise downtown commercial districts (focus on Iowa, Michigan, Ohio, and Wisconsin)	1997–2019	To stabilise and improve the economic vitality of rural downtowns across the United States	Whole of society partnership (National Trust for Historic Preservation, local government, regional government and businesses)	Social determinants of health	Neighbourhood employment rate	Neutral
**Giusti & Maraschin (2017) [84]**	United States; inner city retail area	Assess impacts on urban spatial structure and resident’s socio-economic characteristics	Area-based initiative	Main Street Program; providing resources and practical knowledge to revitalise downtown commercial districts, including rehabilitation of historic buildings, streetscape improvements, incentives to attract new businesses, assisting businesses with financing and permits, revisions of zoning rules, and place marketing	1992–2001	Contribute to inner town regeneration and increase downtown vitality.	Whole of society partnership (National Trust for Historic Preservation, local government, regional government and businesses)	Social determinants of health	Socio-economic pattern (household income); local economic activities (employment)	Neutral
**Lindsay, Lambert, Penn, Hedges, Ortwine & Mei (2013) [85]**	United States; Disadvantaged neighbourhood	Assess impacts on fruit and vegetable purchasing and consumption; and farmers market revenue	Demand-side incentives	Monetary incentive (Farmers Market Fresh Fund Initiative Program) at two local farmers markets in low-income neighbourhoods in San Diego giving additional discounts to recipients of food assistance (SNAP)	2010–2011	Increase the use of federal food assistance at farmers’ markets to increase access to affordable healthy foods and economically support local farmers and social environments	Whole of society partnership (County of San Diego Health and Human Services Agency, University of California San Diego, International Rescue Committee, and local businesses)	Food consumption and purchasing	Fruit and vegetable purchase; fruit and vegetable consumption; self-reported diet quality	Positive
**Baronberg, Dunn, Nonas, Dannefer & Sacks (2013) [86]**	United States; Disadvantaged areas	Assess impacts on fruit and vegetable purchases at farmers market	Demand-side incentives	Monetary incentive (Health Bucks) in New York, offering coupons on purchased fruit and vegetables to recipients of food assistance (SNAP)	2006–2009	Increase the use of federal food assistance at farmers’ markets to increase access to affordable healthy foods and economically support local farmers and social environments	Whole of society partnerships (New York City Department of Health and Mental Hygiene, community organisations and local businesses)	Food consumption and purchasing	Fruit and vegetable spending	Positive
**Young, Aquilante, Solomon, Colby, Kawinzi, Uy & Mallya (2013) [87]**	United States; Disadvantaged neighbourhood	Assess impacts on fruit and vegetable intake and farmers market sales	Demand-side incentives	Monetary incentive (Philly Food Bucks) at local farmers market in low-income areas of Philadelphia giving additional discounts to recipients of food assistance (SNAP)	2010–2011	Increase the use of federal food assistance at farmers’ markets to increase access to affordable healthy foods and economically support local farmers and social environments	Whole of society partnership (Regional government, The Food Trust and local businesses)	Food consumption and purchasing	Fruit and vegetable consumption	Positive
**Freedman, Mattison-Faye, Alia, Guest & Hébert (2014) [88]**	United States; Disadvantaged neighbourhood	Assess impacts on purchases and farmers market revenue	Demand-side incentives	Monetary incentive (Shop n Save) at local farmers market in rural South Carolina giving additional discounts to recipients of food assistance (SNAP)	2012	Increase the use of federal food assistance at farmers’ markets to increase access to affordable healthy foods and economically support local farmers and social environments	Whole of society partnership (Community organisation, university, regional government and local businesses)	Food consumption and purchasing	Purchases at farmers market	Positive
**Gosliner, Hewawitharana, Strochlic, Felix & Long (2022) [89]**	United States; Ethnically diverse state	Assess impacts on fruit and vegetable purchasing and consumption, and food security	Demand-side incentives	Monetary incentive (SNAP) at five local farmers markets in California giving additional discounts to recipients of food assistance	2018	Increase the use of federal food assistance at farmers’ markets to increase access to affordable healthy foods and economically support local farmers and social environments	Public-private partnership (Regional government and local businesses)	Food consumption and purchasing	Household food security; fruit and vegetable consumption	Positive
**Freedman & Kuhns (2018) [90]**	United States; Disadvantaged neighbourhoods	Assess impacts on food shopping and purchasing patterns	Business incentives	New Markets Tax Credit (NMTC) Program, offering tax credits against federal income tax to encourage private investment in target low-income neighbourhoods. A substantial amount went to investment in food businesses	2003–2009	Promote greater investment in operating businesses and real estate projects located in low-income neighbourhoods	Public-private partnership (US Department of Treasury and local businesses)	Food consumption and purchasing; Social determinants of health	Food shopping; food purchasing; supermarket employment	Limited positive
**Farley, Sacks, Dannefer, Johns, Leggat, Lim, Konty & Nonas (2015) [91]**	United States; Disadvantaged neighbourhoods	Assess impact on access to fruit and vegetables; and neighbourhood food retailers	Business incentives	NYC Green Carts Program, expanding the amount of mobile produce vendors in low-income neighbourhoods by granting new permits	2008	To address neighbourhood disparities in the availability and consumption of healthy foods in NYC and positively influence the local food environment.	Public-private partnership (NYC Department of Health and Mental Hygiene and local businesses)	Environmental determinants of health	Neighbourhood fruit and vegetable availability; fruit and vegetable variety; fruit and vegetable quality	Positive
**Li, Cromley, Fox & Horowitz (2014) [92]**	United States; Disadvantaged neighbourhoods	Assess mobile food vendor location and impact on food environment						Food consumption and purchasing	Fruit and vegetable mobile vendor location; fruit and vegetable availability	Neutral
**Lucan, Maroko, Shanker & Jordan (2011) [93]**	United States; Disadvantaged areas	Assess fruit and vegetable vendor availability and spread						Environment determinants of health	Amount of fruit and vegetable vendors; Location of fruit and vegetable vendors	Limited positive
**Hagen & Tennøy (2021) [94]**	Norway; inner city retail area	Assess impacts on (active) travel and city centre vitality	Area-based initiative	Radical street reallocations in the Oslo city centre, including removal of on-street parking spaces, reuse of the spaces for other purposes, physical improvements, activities and events	2017–2019	To create a more vibrant and enjoyable city centre, encouraging public life, business turnover and active travel	Local government	Environmental determinants of health; Physical activity	Mode of transport; active travel; area attractiveness	Limited positive
**Gibbons, Overman & Sarvimäki (2021) [95]**	United Kingdom; Disadvantaged neighbourhoods	Assess impact on employment of local residents in deprived neighbourhoods	Area-based initiative	Single Regeneration Budget; UK government fund allocating money to enhance the quality of life of people in disadvantaged areas. This study has a particular focus on 165 interventions subsidising the building of commercial floor space without direct fiscal incentives to firms.	1994–2002	To bring about multifaceted, economic and social regeneration; enhance the quality of life of local people in deprived areas; and support business development in places that suffered physical decline	Whole of society partnership (UK Government, local governments, training and enterprise councils, voluntary and community organisations, businesses and European Union)	Social determinants of health	Neighbourhood employment rate; Workplace employment	Neutral
**Rhodes, Tyler & Brennan (2005) [96]**	United Kingdom; Disadvantaged neighbourhood	Assess impact on key outcomes related to quality of life	Area-based initiative	Single Regeneration Budget; UK government fund allocating money to enhance the quality of life of people in disadvantaged areas. Interventions include those to improve education and employment opportunities, improve the physical environment and infrastructure, tackling crime, and schemes to support local business competitiveness and marketing initiatives. This study focuses on seven case studies.	1994–2002	To bring about multifaceted, economic and social regeneration; enhance the quality of life of local people in deprived areas; and support business development in places that suffered physical decline	Whole of society partnership (UK Government, local governments, training and enterprise councils, voluntary and community organisations, businesses and European Union)	Social determinants of health	Number of jobs created; number of residents accessing employment through training; number of young people benefiting from personal and social development; number benefiting from community safety initiatives; number of people with access to new cultural facilities; number of people with access to new health facilities	Limited positive
**Olsho, Klerman, Wilde & Bartlett (2016) [97]**	United States; Disadvantaged area	Assess impacts on fruit and vegetable consumption	Demand-side incentives	USDA Healthy Incentives Pilot in Massachusetts, offering 30% rebates on purchased fruit and vegetables to recipients of food assistance (SNAP)	2011–2012	Increase the use of federal food assistance at farmers’ markets to increase access to affordable healthy foods and economically support local farmers and social environments	Whole of society partnership (United States Department of Agriculture, community organisations and local businesses)	Food consumption and purchasing	Fruit and vegetable consumption	Positive

**Table 2:** Information from included grey literature.

Authors (date)	Country; Context	Study aims	Intervention type	Intervention specified	Time frame	Intervention aims	Ownership	Outcome type	Health and wellbeing related outcomes	Intervention impact
**Rhodes, Tyler & Brennan (2007) [22]**	United Kingdom; urban areas	Assess the scheme social, economic and physical outputs, additionality and value for money, and process evaluation	Area-based initiative	Single Regeneration Budget; regeneration fund funding various interventions in selected disadvantaged areas.	1994–2002	To bring about multifaceted, economic and social regeneration; enhance the quality of life of local people in deprived areas; and support business development in places that suffered physical decline	Whole of society partnership (UK Government, local governments, training and enterprise councils, voluntary and community organisations, businesses and European Union)	Social determinants of health; Environmental determinants of health; Physical health	Training and education; employment rate; income; community development; area satisfaction; crime and safety outcomes; self-reported health	Positive
**Brennan, Rhodes & Tyler (1999) [98]**	United Kingdom; urban areas	Assess impacts on cost-effectiveness, wider social achievements and partnership working.	Area-based initiative	Single Regeneration Budget; regeneration fund funding various interventions in selected disadvantaged areas. This study is of three case studies focusing on increasing start-up rate, employment for young people, physical and social regeneration and crime and safety initiatives.	1994–2002	To bring about multifaceted, economic and social regeneration; enhance the quality of life of local people in deprived areas; and support business development in places that suffered physical decline	Whole of society partnership (UK Government, local governments, training and enterprise councils, voluntary and community organisations, businesses and European Union)	Social determinants of health; Environmental determinants of health	Social inclusion, community involvement, impacts on equity	Limited positive
**Brennan, Rhodes, Tyler & Tarling (2000) [99]**	United Kingdom; urban areas	Assess impacts on cost-effectiveness, wider social achievements and partnership working.	Area-based initiative	Single Regeneration Budget; regeneration fund funding various interventions in selected disadvantaged areas. This study is of three case studies focusing on training and education in engineering, town centre regeneration, community development and local employment opportunities.	1994–2002	To bring about multifaceted, economic and social regeneration; enhance the quality of life of local people in deprived areas; and support business development in places that suffered physical decline	Whole of society partnership (UK Government, local governments, training and enterprise councils, voluntary and community organisations, businesses and European Union)	Social determinants of health	Social inclusion, community involvement, impacts on equity	Limited positive
**Deas, Robson, & Bradford (2000) [100]**	United Kingdom; urban areas	Assess impacts on revival of local land and property markets, and economic activity, improvements in social and political outcomes	Area-based initiative	Urban Development Corporations; designated areas with statutory powers and government subsidies to spend on/enable area regeneration and revitalisation initiatives. The study focuses on the three case studies.	1981–1992	To bring land and buildings into effective use, encourage the development of existing and new industry and commerce, create an attractive environment, and ensure housing and social facilities to attract new residents and workers	Private-public partnership (Secretary of State for the Environment and private sector stakeholders)	Social determinants of health	Direct employment generated	Limited positive
**Regeneris Consulting (2015; 2015) [101, 102]**	United Kingdom; urban areas	Assess impacts on economic and social uplift and programme delivery	Area-based initiative	Outer London Fund; delivering funding for initiatives aimed at enhancing place shaping activity and economic growth across outer London’s town centres.	2011–2014	To strengthen the vibrancy and economic growth of high streets and their environs; and improve the quality of local life	Whole of society partnership (Greater London Authority, local authorities, businesses, third sector organisations)	Social determinants of health; Environmental determinants of health	Community floorspace creation; local employment creation; improvement of public realm	Limited positive
**Bourn (1993) [103]**	United Kingdom; urban areas	Assess impacts on physical regeneration and social outcomes	Area-based initiative	Urban Development Corporations; designated areas with statutory powers and government subsidies to spend on/enable area regeneration and revitalisation initiatives, including buying and developing land, environmental improvements, provision of health, educational and community facilities, disbursement of grants to enable private sector commercial, industrial and residential property development, and place marketing. The study focuses on the eight case studies.	1981–1992	To bring land and buildings into effective use, encourage the development of existing and new industry and commerce, create an attractive environment, and ensure housing and social facilities to attract new residents and workers	Private-public partnership (Secretary of State for the Environment and private sector stakeholders)	Social determinants of health; Environmental determinants of health	Investment in housing; investment in social, community and training schemes	Positive
**Esen (2006) [104]**	United States; inner city retail areas	Assess impacts area vitality, physical environment and social outcomes	Area-based initiative	Main Street Program; providing resources and practical knowledge to revitalise downtown commercial districts (focus on Boston)	1995 onwards	To preserve and revitalise economic viability and vitality of historic commercial districts	Whole of society partnership (National Trust for Historic Preservation, local government, regional government and businesses)	Social determinants of health; Environmental determinants of health	Crime rates; liveliness and attractiveness of area; employment for residents; community involvement	Positive
**Ministry of Housing, Communities and Local Government (2020) [105]**	United Kingdom; inner city retail areas	Assess immediate social benefits	Area-based initiative	Open Doors Pilot programme; initiative to bring vacant properties in high streets and town centres into temporary use by local community groups and charitable organisations. The scheme covered business rates and utility bills of the property for landlords, but not rent payments.	2018–2020	Creating socially and economically stronger and more confident communities, and increasing footfall in high streets	Whole of society partnership (Ministry of Housing Communities and Local Government, Meanwhile Foundation, community organisations and local landlords)	Social determinants of health; Environmental determinants of health	Provision and reach of services to people at greater risk of suffering from loneliness	Positive

### Types of interventions

Of the 62 included studies, some evaluated the same intervention, leaving 44 unique interventions (Tables 1 and 2). Several large-scale national interventions that were identified, including the Single Regeneration Budget (UK) [22, 95, 96, 98, 99], Urban Development Corporations (UK) [100, 103], Main Street Program (US) [83, 84, 104], Enterprise Zones (US) [56–63], Empowerment Zone and Enterprise Community (US) [53, 54, 63] and the Federal Healthy Food Financing Initiative (HFFI) (US) [64–68], were delivered at the local or regional level by different stakeholders. The intervention delivery differed by area, particularly when funding was made available for different projects within the intervention remit. Evaluations were therefore only classified as ‘the same’ where they reported on the same setting (e.g. the introduction of a food retail development in Glasgow (UK) [75–77] or a HFFI-funded project in Pittsburgh US) [66, 67]). Notably, the Single Regeneration Budget (UK) [22, 95, 96, 98, 99] is not considered a single intervention in this review due to the evaluations’ focus on different local case studies with different objectives. Four types of interventions were identified: area-based initiatives (n = 17, 23 studies), business improvement districts (n = 5, 8 studies), business or supply-side incentives (n = 16, 25 studies), and demand-side incentives (n = 6; 6 studies) (Table 3). The four types of interventions can broadly be classified as either financial in nature (interventions aimed at financially supporting businesses); policy/regulation related (interventions aimed at modifying the regulatory environment for businesses); physical (interventions aimed at modifying the built environment); or cultural (interventions aimed at strengthening and investing in the local community). Initiatives were typically a mixture of all of these. There is some overlap between area-based initiatives and business incentives, as providing funding to organisations (including businesses) is often part of area-based initiatives. However, area-based initiatives are broader in scope, including areas of work that address social outcomes (e.g. providing education, safety initiatives, targeting vulnerable groups) and the built environment. While some of their work is directly with businesses, they also support regeneration partnerships with local governments and other public bodies, and voluntary and community organisations. This support is not always financial. In the UK (n = 12, 11 interventions in England and one in Scotland), high street or retail elements of area-based initiatives included the development of a new retail, business and leisure centre, building new commercial floor space, schemes to increase local business competitiveness, the introduction of a new food retail developments, and an intervention to fill vacant retail space [22, 73–77, 81, 82, 84, 95, 96, 98–103, 105]. The area-based initiatives identified in the US (n = 4) focused on non-financial business support, storefront improvement grants and infill of empty retail space (withing the broad revitalisation of historic commercial districts) and the introduction of new food retailers [79, 80, 83, 84, 104]. One intervention in Norway focused on street reallocations in a city centre [94]. On the other hand, business incentives are more simply given to companies based on the area they are located in to run their business, with the idea that this will lead to area regeneration and improved social outcomes. These included Enterprise Zones (in different areas in the US) and grants, and tax and zoning incentives for healthy food shops, carts and markets in underserved neighbourhoods (in different areas of the US, and Canada). Included studies of Business Improvement Districts (BIDs) and demand-side healthy food voucher interventions were from the US (evaluations of BIDs from other countries that met the inclusion criteria were not identified). With the exception of BIDs located in inner-city commercial areas, most interventions targeted ‘disadvantaged’ areas in terms of socio-economic characteristics and/or physical condition (Tables 1 and 2). Some identified interventions had explicit health outcomes, as well as outcomes related to business and area revitalisation. This includes all demand-side interventions and several business incentive interventions that aimed to improve availability and access to healthy food in low-access areas or ‘food deserts’.

**Table 3:** Interventions identified in the included studies.

Intervention type	Description	Category	Intervention	Examples
**Area-based initiative**	Policy initiatives aimed at improving economic, social, and environmental outcomes within a (disadvantaged) target area	Financial; Policy/regulation; Physical; Cultural	Regeneration funds	Single Regeneration Budget (UK) [22, 95, 96, 98, 99]; Outer London Fund (UK) [101, 102]
			Place promotion	Main Street Program (US) [83, 84, 104]; Outer London Fund (UK) [101, 102];
			Urban development corporations	Urban Development Corporations (UK) [100, 103]
			Introduction of new retail development under regeneration partnerships	Introduction of new hypermarkets in ‘food deserts’ in Glasgow [75–77], Leeds [73, 74] (UK), and Flint [79, 80] (US); and new retail and leisure centre in London [81, 82] (UK)
			Reallocation of built retail environment	Open Doors Programme (UK) [105]; Street reallocations (Norway) [94]; Main Street Program (US) [83, 84, 104]
**Business improvement district (BID)**	Formal entity of businesses in a defined retail area that uses private funds to invest in the betterment of the area, including physical improvements, sanitation, private security and marketing and place promotion, to increase economic growth and commercial activity.	Financial; Policy/regulation; Physical	BIDs	BIDs in Los Angeles [46–49], Philadelphia [51, 52], New York City [50], Cincinnati [45] (US)
**Business incentives**	Tax incentives and/or grants aimed at facilitating business development and growth in a particular geographic area to meet local needs	Financial; Policy/regulation	Funding for businesses in target areas	Empowerment Zone and Enterprise Community (US) [53, 54, 63]; Federal Healthy Food Financing Initiative (US) [64–68]; subsidising local food market (Canada) [78]
			Tax incentives	Enterprise Zones (US) [56–63]; Food Retail Expansion to Support Health (US) [69–72]; New Markets Tax Credit (US) [90]; introduction of new food retailers (US) [79, 80]
			Advantageous zoning regulation and business permits	Enterprise Zones (US) [56–63]; NYC Green Carts Program (US) [91–93]
			Incentives for job creation	Enterprise Zones (US) [56–63]
**Demand-side incentives**	Monetary incentives for residents to purchase from local businesses	Financial	Discounts and rebates to purchase fresh food from local farmers markets for those on food support	Shop n Save [88]; Farmers Market Fresh Fund Initiative Program [85]; Philly Food Bucks [87]; Healthy Incentives Pilot [97]; California Nutrition Incentive Program [89]; Health Bucks [86] (US)

### Health and wellbeing related outcomes

A minority of evaluations analysed one or more direct health and wellbeing outcomes, including outcomes related to physical health (n = 6), mental health and wellbeing (n = 4), physical activity (n = 2) and food consumption and purchasing (n = 20) (Table 4). Diet-related outcomes predominantly consisted of fruit and vegetable intake, dietary quality, energy intake (assessed through e.g. food frequency questionnaires), and healthy food purchasing and availability. Analysed mental health outcomes were self-reported mental health and psychological wellbeing score (General Health Questionnaire-12).

**Table 4:** Identified health and wellbeing related outcomes in the included studies.

Health and wellbeing related outcomes	Description	Examples
**Physical health**	Direct physical health outcomes and indicators	Self-reported health score [22, 75, 76]
		Body Mass Index [65, 66, 72]
**Mental health and wellbeing**	Direct mental health outcomes and indicators	Self-reported mental health [76]
		Depressive symptoms [81, 82]
		Psychological wellbeing score [75, 81, 82]
**Physical activity**	Physical activity levels	Physical activity levels [65, 82]
		Sedentary behaviour [82]
		Active travel [94]
**Food consumption and purchasing**	Direct and indirect diet and nutrition outcomes	Fruit and vegetable consumption [65, 73–76, 78, 80, 85, 87, 89, 97]
		Dietary intake [64, 65, 69–71, 80]
		Diet quality [65, 66, 73, 74, 85]
		Fast food consumption [65]
		Daily energy intake [66]
		Fruit and vegetable purchasing [71, 85, 86, 88, 90]
		Household food availability [64, 65, 69–71, 80, 89]
**Social determinants of health**	Socio-economic outcomes	Generated employment for local residents [57–59, 61–63, 77, 84, 90, 96, 100–102, 104]
		Training and education access [22, 96, 103]
		(Un)employment rate [50, 53, 56, 62, 63, 83, 95]
		Poverty rate [53, 62, 63]
		Household income [59, 62, 63, 84]
		Social isolation [98, 99, 105]
		Community development [22, 96, 98, 99, 101–104] Crime rate [22, 46, 48, 49, 104]
		Street block robbery [45–49, 52]
		Burglary [46, 47, 52]
		Disorderly conduct [51, 52]
**Environmental determinants of health**	Outcomes related to the physical conditions in which people live	Proximity to closest grocery shop [67, 79]
		Amount of fruit and vegetable retailers [67, 77, 91–93]
		Change in food environment [67, 68, 77, 92]
		Perceived local access to healthy food [65, 66, 78]
		Perceived quality of built environment [94, 96, 101, 102, 104]

More indirect health and wellbeing related outcomes identified in the study reports were those classed as social and environmental determinants of health (Table 4). Outcomes within the social determinants of health (n = 32) include training and employment-related outcomes (e.g. local employment rate, poverty rate, number of jobs created) and community safety outcomes (e.g. street block robbery, violent crimes). Common environmental determinant outcomes (n = 14) were food environment outcomes (e.g. fruit and vegetable availability, proximity to closest grocery shop) (Table 4).

The effectiveness of interventions in achieving the health and wellbeing outcomes of interest was not analysed in depth, as this is beyond the scope of this scoping review. It is important to consider that the interventions were implemented in different contexts, which limits direct comparison. However, as seen in Tables 1 and 2, over two-thirds of evaluations reported positive (n = 21) or limited positive (n = 23) outcomes. In addition, 12 studies reported no significant outcomes and five reported outcomes that are possibly harmful for health and wellbeing. Evidence was classed as “limited positive” if only some findings were positive, but not enough evidence was reported to deem the intervention effective in improving the outcomes of interest. All evaluations of demand-side interventions reported positive outcomes, while 86% of evaluations of area-based initiatives, 63% of BIDs and 55% of business incentives reported positive or limited positive outcomes. All but one study of area-based initiative in the UK reported (limited) positive outcomes (n = 16) but only two out five area-based interventions in the US (Main Street Program in Boston and one study assessing the introduction of a new food retailer in Michigan). Two evaluations reported potential negative health and wellbeing outcomes, while four reported limited negative outcomes. In particular, ‘Enterprise Zone’ business incentives (US) were most frequently found to have no or negative impacts on social determinants of health (73%), including local employment rate. Two evaluations of BIDs (US) reported increases in crime rates.

### Intervention governance

Given the economic regeneration and local business support aims of the evaluated interventions, collaborations between the public and private sector organisations were common. The majority of interventions were either public-private partnerships (PPPs, partnerships between government organisations and the private sector) (n = 22) or whole of society partnerships (n = 19). “Whole of society partnership” is a term used by the World Health Organisation (WHO) and other United Nations organisations for partnerships that include a wide array of relevant stakeholders, including the public sector, voluntary and community organisations, academia, civil society and businesses [106]. One intervention was a public sector intervention and one a partnership between government authorities and community organisations.

Different types of PPPs were identified, with varying levels of involvement and decision-making power for businesses. BIDs are primarily funded and managed by local businesses in a particular commercial area, with more limited involvement and funding from other private, public and voluntary entities. The authors of one study raise concerns that the little public oversight and accountability in the US model of BIDs (different BID models exist in different countries) may lead to conflicts of interests and business misconduct, and harm local democracy [49]. The Urban Development Corporations (UDCs) in the UK were also run by boards predominantly consisting of private sector members. However, they were appointed by and statutorily responsible to the Secretary of State for the Environment, and received significant government subsidies. The underlying idea of these types of partnerships is that private sector leadership and investment is essential in addressing public problems in a way that local governments are not able to [100, 103]. Both US BIDs and UDCs use their collective bargaining power to lobby local government for additional investments in public services and infrastructure, and changes in local regulations, which gives them additional influence over the regeneration process. Other PPPs include joint investments between local government authorities and private investors (e.g. introduction of new food retail developments through regeneration partnerships), or grants and tax benefits for initiatives or activities run by businesses to fulfil (local) government objectives; such as high street revitalisation, increased healthy food availability and providing employment opportunities for residents. An example of a whole of society intervention is the Single Regeneration Budget (UK), which was a partnership between the UK government, local authorities, training and enterprise councils, voluntary organisations, businesses and the European Union, to enhance quality of life and support businesses development in disadvantaged areas.

There are also differences in the types of businesses that are involved in interventions to support retail environments. While some interventions require participation from smaller independent businesses (e.g. food cart owners), others are partnerships with major (inter)national retailers and property development corporations.

### Intervention theories of change

Studies described the mechanisms through which the evaluated interventions were expected lead to health and wellbeing related outcomes, as well as possible reasons (based on theory and empirical findings) why this may not be the case. The detail in which theories of change were described varies. Only 11 studies referred to a specific theoretical framework, including spatial interaction theory [50], crime pattern theory [45], broken window theory [47, 49, 51, 52], defensible space theory [51, 52], routine activities theory [49, 52], and the socio-ecological model for disease prevention [91]. Several themes were created based on the synthesis of data across all included evaluations.

#### Reductionism

Interventions were often criticised for being based on ‘simple’ theories of change that demonstrate a reductionist view of how health and social outcomes are achieved. A very common theory of change in the identified literature was that improvements in diet and food purchasing would follow directly from increasing access and availability to food retail outlets [65–67, 69–77, 80]. Improving food access was not only assumed to reduce diet-related problems, but also to stimulate wider retail-led area regeneration (e.g. by attracting more customers, employees and residents) [75]. Another example that was highlighted in the included studies is the idea that “bringing jobs” to disadvantaged areas (e.g. by providing incentives for businesses development) would lead to a direct improvement in employment outcomes for residents [95]. In fact, most of the evaluations of ‘Enterprise Zones’ (US) did not report any benefits in local employment and poverty rates [55–58, 60–62].

#### Contextual factors

Many authors recognised the importance of context in explaining how and when interventions actually lead to improvements in health and its social and environmental determinants. In fact, one of the main criticisms of interventions and their implementers was that they do not sufficiently take contextual factors into account, leading to limited effectiveness. Several authors highlighted the complexity of health behaviours. For example, whether people show improved dietary outcomes as a result of increased availability of (healthy) food in the local area depends on whether they ‘switch’ to new food retailers, which in turn is determined by factors such as affordability, existing shopping habits, individual and cultural preferences, and access to transport [65–67, 69–77, 80]. Furthermore, Elbel and colleagues argued that *“the density of other food sources such as fast-food restaurants may have a larger effect on obesity*, *especially in low-income communities*.” [71]. As a consequence, a more comprehensive package of population and individual-level interventions was recommended, including interventions addressing food prices, marketing and education. Although, some authors argued that these interventions may have positive impacts on local employment and area image, and can therefore be deemed successful in achieving area regeneration objectives [77].

Other authors critiqued the use of a *“universal approach to downtown revitalisation”* without attention to characteristics of the local area in which it is implemented [83]. This can be the case when national area-based interventions (e.g. the Main Street Program (US) [83, 84], Single Regeneration Budget (UK) [22, 95, 96, 98, 99]) are translated to a variety of different local contexts, where they may not have similar effects on outcomes like job opportunities for residents and local employment rate (e.g. due to a mismatch in requested and available skills in the local population). This challenge was identified both in the UK and the US. In addition to this, it can also be difficult to isolate impacts of multi-component area-based interventions [84, 96, 99, 100]. As Giusti and Maraschin note in their evaluation of area-based initiative the Main Street Program (US), *“it is important to bear in mind that as cities are systems composed of many elements*, *interacting through complex relations*, *it is difficult to clearly identify causes and consequences in the process of change”* [84].

#### Unintended effects

Interventions may also have unintended impacts on health and wellbeing related outcomes and the wider retail environment that are not captured by the theory of change. For example, BIDs often significantly invest in crime reduction to create safer and more attractive environments for business and customers [45–49, 51, 52]. While security measures and area upkeep are typically associated with increased safety, BIDs can also attract criminal activity by increasing footfall in the area [45]. Some authors suggest that BIDs (in the US) could lead to ‘spill-over’ of crime to nearby residential areas, but no empirical evidence for this hypothesis was found [47, 49, 52]. More generally, interventions aimed at revitalising neighbourhoods can have effects on property values and were therefore argued to possibly contribute to gentrification and associated negative mental health outcomes [81].

Several authors pointed out that interventions aimed at incentivising the development of new food retailers not only increase access to fruit and vegetables and other healthy foods, but also to ultra-processed foods. This could explain limited improvements in dietary outcomes that were found in the literature. Besides creating more attractive and vibrant areas [66, 74], the introduction of a new food retail development was also suggested to have possible unintended impacts on the wider food environment, for example by competing with smaller businesses that sell healthy food [67, 79]. Ghosh-Dastidar and colleagues argue that such interventions could consider *“including supports for other stores to maintain or increase their healthy offerings in the face of new supermarkets or doing so instead of introducing new supermarkets*.*”* [67]. However, this is not necessarily the case, as Cummins et al.’s findings in Scotland *“challenge the widespread assumption that the opening of a hypermarket always has an immediate and deleterious effect on the retail structure of an area*.” [77].

#### Extent of investment and implementation

Whether interventions bring about change in health and wellbeing also depends on how the intervention is implemented and the extent to which stakeholders decide to invest in social outcomes. A reported critique of UDCs (UK) and the Empowerment Zone and Enterprise Community Initiative (US) was that despite having explicit goals related to social and community development, actual investment in those priorities was quite limited due to their main focus on economic and commercial development [100, 103]. Authors also described how the effectiveness of some types of interventions is dependent on decisions by independent businesses [91–93]. In an evaluation of the NYC Green Carts Program (US), a business incentive intervention that aims to expand the amount of mobile produce vendors in low-income neighbourhoods, it was suggested that *“a market-driven imperative to locate [food businesses] near potential customers may be in tension with the program objective of increasing access to fruits and vegetables in food deserts”* [92].

#### Equity impacts

The majority of interventions incorporated equity by focusing their efforts towards ‘disadvantaged’ areas, often with low income and high unemployment, or more disadvantaged populations within those areas. The concept of ‘food deserts’ was frequently used in US interventions, where this has been found to be a concern due, among others, to the dispersed geography and more limited access to public transport. However, underserved areas in terms of food retail were also a target for intervention in the UK [75–77]. Questions remain about whether interventions (equally) benefit the most in need. Interventions that provide incentives for buying at local farmers markets in the US ensure this by only being available to those already meeting national criteria for food benefits [85–89, 97]. This is a more targeted approach that considers particular pockets of populations with more disadvantage within underserved areas [73, 77, 79]. Wrigley and colleagues argue that interventions should be designed to target the needs of the local population by balancing *“area-based with targeted individual-based policy responses”* [73]. Some interventions funded through the Single Regeneration Budget (UK) had a targeted approach, focusing on young people, ethnic minorities and drug users [98].

## Discussion

Addressing high street decline and supporting local economic growth is high on the policy agenda in many high income countries [12, 18, 21], particularly following the COVID-19 pandemic [20]. Such interventions may have implications for health and wellbeing through the social and environmental determinants of health [1, 2]. This scoping review identified studies evaluating the health and wellbeing impacts of interventions aimed at revitalising the retail environment and supporting local economic growth. Interventions were categorised in four broad types of interventions: area-based initiatives, BIDs, business incentives, and demand-side interventions. All interventions were financial in nature, but some also included modification of the regulatory environment for businesses and the physical retail environment. Some area-based interventions also included objectives related to community development. The majority of evaluated outcomes are indirectly related to health and wellbeing through the social and environmental determinants, although some evaluation outcomes related to food purchasing and consumption, physical activity and mental health. Aside from interventions introducing new food retailers in underserved areas, none of the identified studies focused on increases in the availability of unhealthy commodities (e.g. fast food outlets, alcohol, tobacco and gambling outlets) in the retail environment. This could be because these outcomes are typically more likely to be evaluated for interventions with a more direct public health focus.

The large majority of interventions were either a PPP or a whole-of-society intervention. Previous literature suggests that there is limited evidence for the effectiveness of PPP in public health promotion interventions, particularly when they involve large corporations from the tobacco, alcohol, food or fossil fuel industries [107]. A concern highlighted in these studies is a conflict of interest between different stakeholders, which may interfere with the implementation of evidence-based public health measures that aim to restrict unhealthy commodities [108]. Given the scope of retail environment interventions, collaboration with the private sector, and some degree of involvement, is necessary. Unlike the case with many health promotion partnerships, the involved businesses do not necessarily directly benefit from health damaging products. However, studies in this review suggest that that trade-offs and conflicts of interest are still relevant, and may influence interventions’ impacts on health and social outcomes regardless of the interventions’ main intended outcomes [47, 49, 53, 92]. This appears to depend on the type of business involvement and the amount of power businesses have over the decision-making process and implementation. Interventions subsidising purchases at local farmers markets may not present any conflicts of interest, as farmers’ goal to sell fruit and vegetables is aligned with the public health objective of improving diets. On the other hand, interventions primarily led by businesses, such as BIDs and UDCs have greater decision-making power over their local area and thus a greater potential to prioritise private interests over social benefit [47, 49, 100]. This does, however, vary by country and the size of the BID. In the US, BIDs are more heavily involved in policy through lobbying [49]. In the UK and Ireland, the powers of BIDs to influence structural change are more limited, but BIDs can influence decisions through partnership with local governments (particularly in Scotland) [109, 110]. In other countries, such as Germany, Sweden and Canada, BIDs have a more operational role with less financial and policy influencing power [111], although they have still been criticised for reinforcing social inequalities [112, 113]. Scholars have called for more comparative research to identify the differential impacts and implications of BIDs internationally [113].

Although evaluating the effectiveness of interventions was outside the scope of this research, we did assess the direction of impacts, as it can inform future research on the topic. About two-thirds of the evaluations reported positive or limited positive broad health and wellbeing outcomes. However, three studies suggest Enterprise Zones in the US may also have negative impacts on employment-related outcomes for the local population, possibly due to a mis-match between the generated employment and local skills and needs. Instead, demand-side interventions aiming to increase access to heathy food for people on benefits were identified as promising in the US. Similar interventions, like the Healthy Start vouchers in the England [114], were not included in this review due to not having an explicit aim to support the local retail environment. This review included area-based initiatives that had multiple regeneration aims (predominantly in the UK) that can have wider impacts on health and wellbeing. Where evaluations of specific retail components were not available, these effects can be hard to separate [84]. The health and wellbeing outcomes reported in this review should thus be considered in the context of evidence of the wider population impacts of area regeneration initiatives. This includes housing regeneration [39, 115] (predominantly reported in UK studies) and improvements in the physical and natural environment, which have been reported to have the potential to improve health behaviours and outcomes in the US, UK and other European countries [33, 34, 115]. One included study assessed the impacts of high street reallocations on transport in a central retail area [94]. Wider area regeneration programmes have been found to have the potential to increase active travel through improvements in connectivity and traffic safety [116], although benefits are possibly unequally distributed [117]. There also potential unintended consequences. Gentrification, and its associated mental health impacts, was highlighted as a possible unintended outcome in one of the included studies assessing an area-based initiative in the UK [81]. Other studies discuss gentrification in the context of urban regeneration (and BIDs [118]) in the UK [119], US [120], and the ‘Global North’ [115, 119, 121]. The displacement from regenerated areas has financial and (mental) health implications for individuals, and changes neighbourhoods and communities, including their demographic composition, social fabric, access to transport, and amenities and services [121]. While direct health impacts are challenging to measure, negative impacts have been found to disproportionately affect low-income and minority groups in the US [120, 122] and a number of other countries, including the UK, Canada, Australia, Spain and other European countries [119]. However, this appears to depend on pre-existing inequalities, which will differ by urban context and country [122], and the extent to which interventions are inclusive and participatory [119].

This raises the question about how the Health in All Policies vision, which calls for the inclusion of public health objectives in every government policy, is effectively applied to policies to encourage economic growth and retail environment support. A finding of this review is that the mechanisms through which many retail environment interventions are expected to impact on health and related social and environmental outcomes are simplistic. Theories of change tended to not capture the real-world context in which interventions are implemented. Authors of the included studies suggested that this affected the extent to which interventions were successful in achieving health and wellbeing outcomes. It should be noted that 15 of the included evaluations did not have a robust study design. In general, health and wellbeing outcomes were often not the main intended outcomes of the interventions and their implementers, which tended to focus on economic growth and business development. Where health and wellbeing outcomes were taken into account, these were mostly indirect health outcomes, and a lack of prioritisation of these objectives through investment and implementation was at times a barrier. An added layer of complexity is that interventions were implemented in different contexts, although the majority of interventions took place in the US and UK. Area-based initiatives predominantly took place in the UK (England and Scotland), and findings may thus have limited relevance to other contexts. Other interventions took place in the US, which may be different from similar interventions in Europe and other continents due to its more dispersed and suburban geography and different policy environment. Countries outside North America and Europe were not represented in the identified literature.

The public health community is interested in the possible (unintended) health impacts of interventions aimed at stimulating local economic growth. However, it has generated relatively little evidence to understand how retail environments can be made more economically viable in ways that also directly support population health. This lack of understanding about a key policy priority may be a barrier to working with policy-makers to create healthier high streets. The evidence found in this scoping review suggests that health and wellbeing are often not meaningfully integrated into the design of revitalisation interventions, for example through a clear theory of change that addresses the complex systems in which these interventions take place. Rhodes and colleagues argue that for large-scale interventions, a comprehensive theory of change is not only necessary at the national level, but also for implementation at local level [96]. They also see a lack of understanding of theories of change as a barrier to identifying effective policy interventions, thus hampering high-quality evaluations [96]. When it comes to implementation, interventions may benefit from choosing the right partners, setting appropriate conditions for receiving funds and concessions, and integrating desired health and wellbeing outcomes in the evaluation framework. Recently, researchers have called for a shift in thinking from Health *in* all Policies to Health *for* all Policies [123]. This subtle change in wording seeks to emphasise what health policy can contribute to other sectors, focusing on co-benefits in both directions, and could encourage greater collaboration between health, economic and other sectors.

### Limitations

To our knowledge this is the first review scoping the literature on retail environment revitalisation interventions and public health. The focus was mapping different types of interventions and the ways they are theorised to influence health and wellbeing outcomes. The review did therefore not focus on the methodological quality of studies and did include several reports that were not peer-reviewed. Study selection was predominantly performed by one author, with 10% second screening at title and abstract and full text screening phases. We searched five academic databases, including those focusing on health (MEDLINE and Embase) and other disciplines (Web of Science, EconLit, Social Policy and Practice), as well as Google and Google Scholar search engines and Open Grey. While studies were identified using various methods and sources, relevant studies (particularly in the grey literature) were possibly missed, for example because of the use of different terminology for relevant interventions. We did for example not use the word ‘place’ in our search, which would have captured a wider scope of studies but in a way that greatly reduced the specificity of our search strategy. We may have also missed relevant studies by not searching additional databases, such as Scopus and business databases. Studies were only included if they were available in English, which likely contributed to the limited geography represented in the sample, particularly when it comes to grey literature. The lack of representation of low and middle income countries in the sample also reflects differences in challenges when it comes to the local retail environment. The decline of the traditional high street, and interventions to counter this trend, are typically written about in the context of high income countries. At the same time, the context between (and within) included countries also varies considerably. While this does not directly impact on our primary aims of classifying interventions and their reported outcomes, it does warrant caution about the interpretation of theories of change and study outcomes, and their transferability to other countries and contexts. In addition, it was challenging to determine whether interventions meet inclusion criteria, particularly when interventions have broad urban regeneration aims. We therefore included interventions if they had a significant and explicit focus on supporting local retail environments and businesses. As study quality was not reviewed, this scoping review did not include an in-depth analysis of study findings. Future research could focus on determining the types of revitalisation interventions that are most likely to benefit health and wellbeing.

## Conclusion

Our social and physical environments influence our health. Interventions that aim to revitalise high street retail environments and support local economic growth therefore also influence health and wellbeing outcomes. This review identified four main types of such interventions. Generally, studies tended to evaluate the interventions’ effects on social and environmental determinants of health, or health behaviours (particularly relating to food consumption and purchasing) rather than direct health outcomes. Theories of change were typically reductionist and failed to acknowledge the complexity of behaviour change. In some cases, health and social outcomes were not prioritised to the same extent as economic outcomes. This could possibly explain the mixed results when it comes to intervention effectiveness in improving health and wellbeing, although interventions may have been effective in supporting the local economy. It is difficult to formulate a policy priority based on the findings of this review, given the few studies evaluating direct health outcomes, different intervention contexts and the limited underpinning theories of change. It appears important that health and wellbeing outcomes are explicitly integrated in retail environment interventions at the design, implementation and evaluation stage, taking into account the complex systems in which interventions take place. This requires collaboration between different departments and sectors.

## Supporting information

S1 Checklist PRISMA extension for Scoping Reviews (PRISMA-ScR) checklist. (DOCX)

S1 Appendix Search strategy. (DOCX)

S1 Dataset Data charting file. (XLSX)
