# Supplementary material for: High street retail environment interventions and their theorised impacts on health and wellbeing: A scoping review
Source: PLoS One. 2024 Nov 14;19(11):e0312826. doi: 10.1371/journal.pone.0312826 (PMC11563429; doi:10.1371/journal.pone.0312826)
Supplement: S1 Appendix — (DOCX) [file pone.0312826.s002.docx]

**Supporting information S1: Search strategy**

**Database: MedLine**

Date: 01/11/2022

| ("urban regeneration" or "urban renewal" or revitalisation or "social planning" or "city planning" or "urban planning" or "town centre" or "high street" or retail or "food environment" or "area based initiative*").mp. [mp=title, book title, abstract, original title, name of substance word, subject heading word, floating sub-heading word, keyword heading word, organism supplementary concept word, protocol supplementary concept word, rare disease supplementary concept word, unique identifier, synonyms] | 21996 |
| --- | --- |
| (health or "health inequalit*" or "heath equity" or "mental health" or wellbeing or "physical activity" or "active travel" or diet).mp. [mp=title, book title, abstract, original title, name of substance word, subject heading word, floating sub-heading word, keyword heading word, organism supplementary concept word, protocol supplementary concept word, rare disease supplementary concept word, unique identifier, synonyms] | 3872369 |
| social planning/ or city planning/ or environment design/ or urban renewal/ | 13084 |
| health/ or population health/ or public health/ or "social determinants of health"/ or exp socioeconomic factors/ | 606545 |
|  |  |
| (evaluation or intervention or "impact assessment").mp. [mp=title, book title, abstract, original title, name of substance word, subject heading word, floating sub-heading word, keyword heading word, organism supplementary concept word, protocol supplementary concept word, rare disease supplementary concept word, unique identifier, synonyms] | 2476197 |
| 1 or 3 | 28373 |
| 2 or 4 | 4091366 |
| 5 and 6 and 7 | 2510 |

**Database: EconLit**

Date: 01/11/2022

| ("urban regeneration" or "urban renewal" or revitalisation or "social planning" or "city planning" or "urban planning" or "urban design" or "town centre" or "high street" or retail or "food environment" or "area based initiative*" or ABI or zoning).mp. [mp=heading words, abstract, title, country as subject] | 22696 |
| --- | --- |
| (health or "public health" or "health inequalit*" or "heath equity" or "mental health" or wellbeing or "physical activity" or "active travel" or diet or "social determinants" or "social factor*" or "socio-economic status").mp. [mp=heading words, abstract, title, country as subject] | 130982 |
| (evaluation or intervention or impact assessment).mp. [mp=heading words, abstract, title, country as subject] | 53375 |
| 1 and 2 and 3 | 62 |

**Data base: Embase**

Date: 01/11/2022

| ("urban regeneration" or "urban renewal" or revitalisation or "city planning" or "urban planning" or "town centre" or "high street" or retail or "food environment" or "area based initiative*").mp. [mp=title, abstract, heading word, drug trade name, original title, device manufacturer, drug manufacturer, device trade name, keyword heading word, floating subheading word, candidate term word] | 21033 |
| --- | --- |
| city planning/ or environment design/ or urban renewal/ or planning/ | 50027 |
| health/ or public health/ or "social determinants of health"/ or exp socioeconomic factors/ or health equity/ | 1593028 |
| (health or "health inequalit*" or "heath equity" or "mental health" or wellbeing or "physical activity" or "active travel" or diet).mp. [mp=title, abstract, heading word, drug trade name, original title, device manufacturer, drug manufacturer, device trade name, keyword heading word, floating subheading word, candidate term word] | 5591294 |
| (evaluation or intervention or "impact assessment").mp. [mp=title, abstract, heading word, drug trade name, original title, device manufacturer, drug manufacturer, device trade name, keyword heading word, floating subheading word, candidate term word] | 3469224 |
| 1 or 2 | 67811 |
| 3 or 4 | 6118459 |
| 5 and 6 and 7 | 5689 |
| Filter: Article  (Excluding Conference Abstracts, Reviews, Articles in Press, Conference Papers, Editorials, Notes and Preprints) | 2646 |

**Database: Web of Science**

Date: 31/10/2022

| ((ALL=("urban regeneration" or "urban renewal" or revitalisation or "city planning" or "urban planning" or "town centre" or "high street" or retail or "food environment" or "area based initiative*")) AND ALL=(health or "health inequalit*" or "heath equity" or "mental health" or wellbeing or "physical activity" or "active travel" or diet)) AND ALL=(evaluation or intervention or "impact assessment") | **3789** |
| --- | --- |
| Filter: Article  (Excluding Review Articles, Proceeding Papers, Early Access, Editorials, Meeting Abstracts, Book Chapters, Data Papers, News Items, Notes, Corrections and Retracted Publications) | 3789 |

**Database: Social Policy and Practice**

Date: 31/10/2022

| (“urban regeneration" or "urban renewal" or revitalisation or "social planning" or "city planning" or "urban planning" or "town centre" or "high street" or retail or "food environment" or "area based initiative*" or ABI).mp. [mp=abstract, title, publication type, heading word, accession number] | 7833 |
| --- | --- |
| (health or "public health" or "health inequalit*" or "heath equity" or "mental health" or wellbeing or "physical activity" or "active travel" or diet).mp. [mp=abstract, title, publication type, heading word, accession number] | 136209 |
| (evaluation or intervention or "impact assessment").mp. [mp=abstract, title, publication type, heading word, accession number] | 58681 |
| 1 and 2 and 3 | 163 |

**TOTAL STUDIES IDENTIFIED: 9170**

**AFTER REMOVAL OF DUPLICATES (n=2508): 6662**

**Additional (grey) literature**

**Search engines: Google Scholar, Google and Open Grey database**

Search terms:

- High street/town centre/main street/urban AND regeneration/revitalisation/renewal
- Retail-led/commercial AND urban regeneration/revitalisation/renewal

Once specific interventions were identified, these were specifically searched for with the key word evaluation.

**Websites: UK Government, Scottish Government, Welsh Government, Greater London authority, Public Health Scotland**

Search Terms included:

- High street
- Revitalisation
- Regeneration
- Town centre
- Business support
- Business improvement districts
- Names of specific identified interventions
